# U-Shaped Association between Plasma Manganese Levels and Type 2 Diabetes

**DOI:** 10.1289/EHP176

**Published:** 2016-06-03

**Authors:** Zhilei Shan, Sijing Chen, Taoping Sun, Cheng Luo, Yanjun Guo, Xuefeng Yu, Wei Yang, Frank B. Hu, Liegang Liu

**Affiliations:** 1 Department of Nutrition and Food Hygiene, Hubei Key Laboratory of Food Nutrition and Safety, and; 2 MOE Key Lab of Environment and Health, School of Public Health, Tongji Medical College, Huazhong University of Science and Technology, Wuhan, China; 3 Division of Endocrinology, Department of Internal Medicine, Tongji Hospital, Tongji Medical College, Huazhong University of Science and Technology, Wuhan, China; 4 Department of Nutrition, and; 5 Department of Epidemiology, Harvard T.H. Chan School of Public Health, Boston, Massachusetts, USA

## Abstract

**Background:**

Manganese is both an essential element and a known toxicant, and it plays important roles in many mechanisms in relation to type 2 diabetes (T2D). However, epidemiological studies of this relationship are rare.

**Objective:**

We investigated the association between plasma manganese and newly diagnosed T2D as well as whether the association could be modified by manganese superoxide dismutase (MnSOD) polymorphisms.

**Methods:**

We conducted a case–control study of 3,228 participants in China: 1,614 T2D patients and 1,614 controls. Concentrations of plasma magnesium were measured, and all participants were genotyped for the MnSOD Val16Ala polymorphism (rs4880).

**Results:**

A U-shaped association was observed between plasma manganese and T2D, with increased odds ratios (ORs) in relation to either low or high plasma manganese levels. Compared with the middle tertile, the multivariate-adjusted ORs [95% confidence intervals (CIs)] of T2D associated with the lowest tertile and the highest tertile of plasma manganese were 1.89 (1.53, 2.33) and 1.56 (1.23, 1.97), respectively. In spline analysis, the U-shaped association was consistently indicated, with the lowest odds of T2D at the plasma manganese concentration of 4.95 μg/L. Minor allele frequencies (C allele) of the MnSOD Val16Ala polymorphism (rs4880) in the normal glucose tolerance (NGT) and the T2D groups were 13.57% and 14.50%, respectively. The MnSOD rs4880 polymorphism was not associated with T2D, and no interaction was found between plasma manganese and the MnSOD rs4880 polymorphism in relation to T2D.

**Conclusions:**

Our results suggested a U-shaped association between plasma manganese and T2D; both low and high levels of plasma manganese were associated with higher odds of newly diagnosed T2D. The U-shaped association was not modified by the MnSOD rs4880 polymorphism.

**Citation:**

Shan Z, Chen S, Sun T, Luo C, Guo Y, Yu X, Yang W, Hu FB, Liu L. 2016. U-shaped association between plasma manganese levels and type 2 diabetes. Environ Health Perspect 124:1876–1881; http://dx.doi.org/10.1289/EHP176

## Introduction

Manganese is an essential micronutrient required for normal carbohydrate, lipid, and protein metabolism (Aschner and Aschner 2005). Manganese is involved in normal immune functions, bone growth, regulation of blood glucose, and cellular energy, and it is a key component of manganese superoxide dismutase (MnSOD) (Aschner and Aschner 2005). MnSOD is a major antioxidant owing to its localization in the mitochondrial matrix, and it plays a critical role in protecting mitochondria and islets from elevated levels of reactive oxygen species (ROS) (Chen et al. 2005; Fridovich 1995), which may serve as an important trigger of insulin resistance and type 2 diabetes (Anderson et al. 2009; Hoehn et al. 2009; Houstis et al. 2006). Despite its essentiality, at excessive levels, manganese is toxic to humans, particularly to the central nervous system (CNS) (Guilarte 2010; Sidoryk-Wegrzynowicz and Aschner 2013), which plays an important role in glucose homeostasis and type 2 diabetes (T2D) (Schwartz et al. 2013).

In animal models, several studies have elucidated that insufficient levels of dietary manganese could result in suboptimal levels of MnSOD activity (Burlet and Jain 2013; Lee et al. 2013), lower insulin secretion (Baly et al. 1985), and reduced glucose uptake and metabolism (Baly et al. 1990). Consistent with the aforementioned findings, manganese supplementation enhanced MnSOD activity and protected against T2D and diabetes complications (Burlet and Jain 2013; Lee et al. 2013). However, with limited sample populations, several epidemiologic studies have yielded inconsistent associations between manganese levels and T2D (Kazi et al. 2008; Koh et al. 2014; Rambousková et al. 2013). As a transition metal, manganese itself is an oxidant at high concentrations, and it appears to be involved in oxidative damage and mitochondrial dysfunction, which have been implicated in the development of T2D (Lowell and Shulman 2005; Maechler and Wollheim 2001). Although excessive manganese has been reported to be associated with neurodevelopmental and neurological disorders (Claus Henn et al. 2010; Guilarte 2010; Sidoryk-Wegrzynowicz and Aschner 2013), epidemiologic evidence regarding the association between excessive manganese and T2D has not been reported.

Both the MnSOD gene and levels of manganese could affect the activity of MnSOD (Bresciani et al. 2013). The MnSOD Val16Ala polymorphism (rs4880), in exon 2 of the human MnSOD gene located on chromosome 6q25, is considered the most interesting polymorphism in the MnSOD gene because the substitution of the T allele for the C allele could result in reduced MnSOD activity and less-efficient transport of MnSOD into the mitochondrial matrix (Shimoda-Matsubayashi et al. 1996; Sutton et al. 2003). The MnSOD Val16Ala polymorphism has been shown to be associated with diabetes and diabetic complications, but the findings are inconsistent (Katakami et al. 2014; Möllsten et al. 2007, 2009; Tian et al. 2011).

To our knowledge, no study has examined the association of both low and high levels of manganese with T2D in humans, and it is unclear whether this association differs according to MnSOD genetic variations. We therefore performed a large case–control study to investigate the association between plasma manganese and newly diagnosed T2D as well as whether the association is modified by the MnSOD Val16Ala polymorphism.

## Methods

### Study Population

The study population consisted of 3,228 participants: 1,614 newly diagnosed T2D patients and 1,614 normal glucose tolerance (NGT) individuals. The patients with newly diagnosed T2D were consecutively recruited from those attending for the first time the outpatient clinics of the Department of Endocrinology, Tongji Medical College Hospital, Wuhan, China, from January 2009 to December 2011. Concomitantly, we recruited healthy NGT individuals who were frequency-matched by age (± 5 years) and sex with patients from an unselected population undergoing a routine health check-up in the same hospital. The inclusion criteria of NGT and newly diagnosed T2D were age ≥ 30 years, body mass index (BMI) < 40 kg/m^2^, no history of a diagnosis of diabetes, and no history of receiving pharmacological treatment for hyperlipidemia or hypertension. Patients with clinically significant neurological, endocrinological, or other systemic diseases, as well as those with acute illness and chronic inflammatory or infective diseases, were excluded from the study. All of the participants enrolled were of Chinese Han ethnicity. The participants provided written informed consent to take part in the study, and they did not take any medication known to affect glucose tolerance or insulin secretion before participation. The study was approved by the ethics committee of the Tongji Medical College.

### Assessment of NGT and T2D

The definitions of T2D met the diagnostic criteria recommended by the World Health Organization in 1999 (Alberti and Zimmet 1998). T2D was diagnosed when fasting plasma glucose (FPG) ≥ 7.0 mmol/L and/or 2-hr post-glucose load ≥ 11.1 mmol/L. An FPG concentration *<* 6.1 mmol/L and a 2-hr oral glucose tolerance test (OGTT) plasma glucose concentration *<* 7.8 mmol/L was considered NGT.

### Body Composition and Blood Parameters

Demographic and health information were collected via a questionnaire; this information included age, sex, current smoking status, current alcohol consumption, physical activity level (hours per week), history of disease (hypertension and hyperlipidemia), and family history of diabetes. Height (m) and weight were measured using standardized techniques. BMI was calculated as weight divided by the square of height (kg/m^2^). After a 10-hr overnight fast, all participants underwent a 75-g OGTT, and venous blood samples were collected at 0 and 2 hr for determination of FPG, OGTT2h, fasting plasma insulin (FPI), total cholesterol (TC), triglycerides (TG), high-density lipoprotein cholesterol (HDL-C), and low-density lipoprotein cholesterol (LDL-C). The homoeostasis model assessment insulin resistance (HOMA-IR) score was computed using the following formula: FPI (milliunits/liter) × FPG (millimoles/liter)/22.5. The index of the HOMA of β-cell function (HOMA-β) was calculated as (20 × FPI)/(FPG – 3.5) (Matthews et al. 1985).

### Measurement of Plasma Manganese Concentrations

Plasma manganese concentrations were measured using inductively coupled plasma mass spectrometry (ICP-MS) (Agilent 7700 Series) in the MOE Key Lab of Environment and Health at the School of Public Health at Tongji Medical College of Huazhong University of Science and Technology. Samples from the T2D and NGT groups were randomly assayed. For quality assurance, the CRMs (certified reference materials) ClinChek No. 8883 and No. 8884 human plasma controls were used. For No. 8883, we determined a concentration of 6.52 ± 0.28 μg/L (certified: 6.72 ± 1.34 μg/L), and for No. 8884, we measured 17.5 ± 0.51 μg/L (certified: 16.9 ± 3.4 μg/L). The intra-assay and inter-assay coefficients of variation of plasma manganese were both < 5%. All participants had plasma manganese levels above the detection limit (0.001 μg/L).

### Genotyping

The MnSOD polymorphism rs4880 was genotyped using an allelic discrimination assay-by-design TaqMan method on an ABI 7900HT PCR system (Applied Biosystems). The primers and the labeled oligonucleotide probes were designed and supplied by Applied Biosystems. The TaqMan genotyping reaction was amplified (50°C for 2 min, 95°C for 10 min, followed by 40 cycles of 92°C for 15 sec and 60°C for 1 min), and the end point fluorescent readings were performed using ABI 7900HT data collection and analysis software v.2.2.1 (SDS 2.2.1). The genotype success rate was 98.12% for rs4880, and Hardy–Weinberg equilibrium tests were performed.

### Statistical Analysis

General demographic and laboratory characteristics were summarized as the mean ± standard deviation (SD) or as the median with interquartile range (IQR), depending on the normality of the continuous variables, or they were summarized as numbers with proportions for categorical variables. To test for differences of characteristics among different glucose regulation status, continuous variables were compared using one-way analysis of variance (ANOVA), and a χ^2^ test was used for categorical variables. For calculation of the odds ratio (OR) for T2D, plasma manganese concentrations were treated as continuous variables and were categorized in tertiles according to the NGT group: tertile 1, ≤ 4.21 μg/L, tertile 2, 4.21–6.84 μg/L, and tertile 3, ≥ 6.84 μg/L. Binary logistic regression analysis was used to assess the associations of T2D with plasma manganese concentrations. ORs and 95% confidence intervals (CIs) were adjusted for known risk factors for T2D, including age, sex, BMI, current smoking status, current alcohol consumption, physical activity levels (never or rare, 1 to 2, 3 to 4, ≥ 5 hr/week), hypertension, family history of diabetes, plasma iron, plasma copper, and plasma selenium. Stratified analyses were conducted by age, sex, BMI, current smoking status, current alcohol consumption, physical activity levels, hypertension, and family history of diabetes. To further explore the potential nonlinearity of the relationship between plasma manganese concentration and T2D, a logarithmic transformation was used to improve the normality of the plasma manganese distributions, and we used restricted cubic splines with 4 knots at the 20th, 40th, 60th, and 80th percentiles of ln(plasma manganese concentration), excluding values outside the 5th and 95th percentiles (Stata version 12; Stata Corp). The distributions of the rs4880 genotypes were analyzed for deviation from Hardy–Weinberg equilibrium using a likelihood ratio test. Binary logistic regression analysis was also used to assess the associations of T2D with rs4880 polymorphisms in log-additive and dominant models. In addition, we examined the association between plasma manganese concentration (tertiles) and T2D stratified by rs4880 polymorphisms (CC, CT, CC + CT vs. TT genotypes), as well as the association between rs4880 polymorphisms and T2D according to plasma manganese tertiles.

To test the interaction between plasma manganese concentrations and rs4880 polymorphisms in association with T2D, we introduced a multiplicative interaction term of genotypes (CC, CT, CC + CT vs. TT genotypes) and plasma manganese tertiles as continuous variables and added these variables to the aforementioned multivariate model. A likelihood ratio test with one degree of freedom was used to assess the significance of the interaction with a comparison of the likelihood scores of the two models with and without the interaction term. All data analyses were performed using SAS 9.1 (SAS Institute Inc., Cary, NC, USA). The *p*-values presented are two-tailed with a significance level of 0.05.

## Results

General anthropometric and metabolic characteristics of the 3,228 participants (1,614 T2D and 1,614 NGT) are summarized in Table 1. Compared with control subjects, the individuals with T2D had higher BMI, greater prevalence of family history of diabetes and hypertension, and higher levels of TC, TG, FPG, FPI, and OGTT2h. Lower HOMA-β and higher HOMA-IR were observed in the T2D group than in the controls. Medians (IQR) of plasma manganese concentration were 5.26 μg/L (3.67–8.33) for the NGT group and 4.37 μg/L (2.73–7.62) for the T2D group. Minor allele frequencies (C allele) of rs4880 in the NGT and T2D groups were 13.57% and 14.50%, respectively. The genotype distributions of rs4880 were in Hardy–Weinberg equilibrium for both the T2D (*p* = 0.39) and NGT (*p* = 0.80) groups.

**Table 1:** Anthropometric and metabolic characteristics of NGT and T2D groups.

Parameters	NGT	T2D	*p-*Value
*n*	1,614	1,614
Age (years)	54.69 ± 10.41	52.54 ± 9.79	0.06
Male, *n* (%)	910 (56.38)	922 (57.12)	0.63
BMI (kg/m^2^)	23.56 ± 3.44	24.96 ± 3.61	< 0.01
Current smoker, *n* (%)	490 (30.36)	477 (29.55)	0.03
Current drinker, *n* (%)	450 (27.88)	462 (28.62)	0.01
Family history of diabetes, *n* (%)	114 (7.06)	311 (19.27)	< 0.01
Hypertension, *n* (%)	394 (24.41)	565 (35.01)	< 0.01
Fasting plasma glucose (mmol/L)	5.27 ± 0.54	9.44 ± 3.13	< 0.01
OGTT2h (mmol/L)	6.43 ± 0.92	17.08 ± 5.03	< 0.01
Hemoglobin A1c	5.58 ± 0.44	8.60 ± 3.43	< 0.01
Triglycerides (mmol/L)	1.16 (0.84–1.65)	1.44 (1.05–2.08)	< 0.01
Total cholesterol (mmol/L)	4.39 (3.57–5.14)	4.61 (3.98–5.32)	< 0.01
Fasting plasma insulin (μU/mL)	7.62 (4.67–11.75)	8.88 (5.83–13.36)	< 0.01
HOMA-β	80.12 (50.36–120.39)	35.32 (17.69–61.04)	< 0.01
HOMA-IR	1.79 (1.11–2.79)	3.63 (2.32–5.33)	< 0.01
Manganese (μg/L)	5.26 (3.67–8.33)	4.37 (2.73–7.62)	< 0.01
MnSOD SNP rs4880			0.38
Allele C	438 (13.57)	468 (14.50)
Allele T	2,790 (86.43)	1,184 (85.50)
CC genotype	30 (1.86)	38 (2.35)	0.50
CT genotype	378 (23.42)	392 (24.29)
TT genotype	1,206 (74.72)	1,184 (73.36)
Abbreviations: BMI, body mass index; HOMA-β, homeostasis model assessment of beta cell function; HOMA-IR, homeostasis model assessment of insulin resistance; IGR, impaired glucose regulation; MnSOD, manganese-dependent mitochondrial superoxide dismutase; NGT, normal glucose tolerance; OGTT2h, 2-hr post-glucose load; SNP, single nucleotide polymorphism; T2D, type 2 diabetes mellitus. Data are presented as number (percentage) for categorical data, mean (standard deviation) for parametrically distributed data or median (interquartile range) for nonparametrically distributed data.			

**Figure 1:**
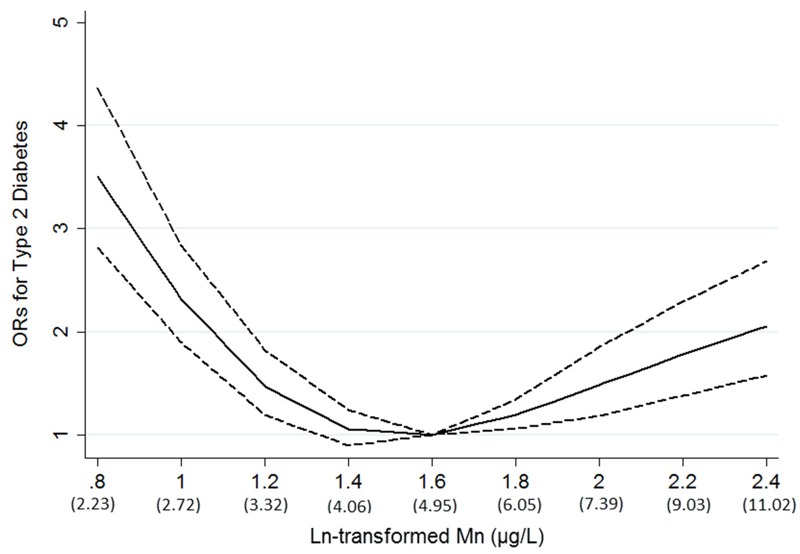
Adjusted odds ratios (ORs; solid line) and 95% confidence intervals (dashed line) for type 2 diabetes (T2D) by ln-transformed plasma manganese (Mn) concentrations. Results were adjusted for age, sex, body mass index, current smoking status, current alcohol drinking status, physical activity, family history of diabetes, hypertension, plasma iron, plasma copper, and plasma selenium.

Table 2 presents logistic regression results for T2D associated with the levels of plasma manganese concentrations categorized into tertiles according to the distribution in controls. Compared with the middle tertile, the multivariate-adjusted ORs (95% CIs) of T2D associated with the lowest tertile and the highest tertile of plasma manganese were 1.89 (95% CI: 1.53, 2.33) and 1.56 (95% CI: 1.23, 1.97), respectively. In the spline regression analysis, the nonlinear spline terms were statistically significant (*p* < 0.01 for nonlinearity); a U-shaped association was observed between plasma manganese and T2D, with the lowest odds of T2D at the plasma manganese concentration of 4.95 μg/L (Figure 1). In stratified analyses by age, sex, BMI, current smoking status, current alcohol consumption, physical activity, family history of diabetes, and hypertension, the U-shaped association was consistently observed in all subgroups (Table 3), and significant interactions were found between plasma manganese concentration and age (*p* < 0.01) as well as physical activity (*p* < 0.01). The Mn-diabetes associations for low and high levels of plasma manganese were stronger in participants ≥ 55 years old than in participants < 55 years old. The association with low levels of plasma manganese was stronger than with high levels in the group that did not perform physical activity, whereas the opposite was observed in the group that did perform physical activity.

**Table 2:** Association of plasma manganese concentrations with T2D.

Variables	Tertiles of plasma manganese concentrations (μg/L)		
	1 (Lowest) ≤ 4.21 μg/L	2 4.21–6.84	3 (Highest) ≥ 6.84 μg/L
*n*(NGT/T2D)	538/780	541/377	535/457
Crude	2.08 (1.75, 2.47)	1	1.23 (1.02, 1.47)
Model 1	2.08 (1.73, 2.49)	1	1.28 (1.05, 1.55)
Model 2	2.15 (1.75, 2.63)	1	1.54 (1.23, 1.92)
Model 3	1.89 (1.53, 2.33)	1	1.56 (1.23, 1.97)
Model 1, adjusted for age, sex, and body mass index. Model 2, adjusted for Model 1, current smoking status, current alcohol drinking status, physical activity, family history of diabetes, and hypertension. Model 3, adjusted for Model 2, plasma iron, plasma copper, and plasma selenium.			

**Table 3:** Adjusted ORs for plasma manganese levels associated with T2D in subgroups.

Groups	Tertiles of plasma manganese concentration (μg/L)			*p*-Value for interaction
	1 (Lowest)	2	3 (Highest)	
Sex
Male	2.14 (1.62, 2.83)	1	1.28 (0.96, 1.72)	0.06
Female	2.31 (1.69, 3.17)	1	2.06 (1.47, 2.90)
Age
< 55	1.81 (1.34, 2.45)	1	1.30 (0.94, 1.79)	< 0.01
≥ 55	2.45 (1.80, 3.33)	1	1.77 (1.28, 2.45)
BMI
< 24	2.17 (1.62, 2.90)	1	1.47 (1.07, 2.01)	0.45
≥ 24	2.26 (1.70, 3.02)	1	1.67 (1.24, 2.26)
Physical activity
No or rare	1.97 (1.52, 2.56)	1	1.37 (1.02, 1.82)	< 0.01
Yes	1.55 (1.06, 2.25)	1	2.11 (1.38, 3.22)
Current smoking
Yes	2.21 (1.50, 3.26)	1	1.18 (0.79, 1.75)	0.21
No	2.22 (1.74, 2.84)	1	1.76 (1.36, 2.29)
Current drinking
Yes	1.84 (1.22, 2.78)	1	1.27 (0.84, 1.93)	0.49
No	2.36 (1.85, 3.00)	1	1.73 (1.34, 2.24)
Family history of diabetes
Yes	1.95 (1.09, 3.49)	1	1.31 (0.71, 2.43)	0.73
No	2.29 (1.84, 2.86)	1	1.62 (1.29, 2.05)
Hypertension
Yes	2.62 (1.81, 3.80)	1	1.42 (0.97, 2.10)	0.12
No	2.07 (1.61, 2.66)	1	1.66 (1.27, 2.15)
Adjusted for age, sex, body mass index, current smoking status, current alcohol drinking status, physical activity, family history of diabetes, hypertension, plasma iron, plasma copper, and plasma selenium.				

There was no significant association between rs4880 and T2D in either the main analysis or in analyses stratified by age, sex, BMI, physical activity, or plasma manganese levels (see Tables S1 and S2). The U-shaped Mn-diabetes association was observed in every rs4880 genotype group, and no interaction was found between plasma manganese and rs4880 in relation to T2D (*p* for interaction = 0.54, see Table S3).

## Discussion

To our knowledge, this was the first population-based study showing that the association between plasma manganese and T2D followed a U-shaped curve; both low and high levels of plasma manganese were associated with higher odds of newly diagnosed T2D. In addition, the U-shaped association was not modified by the MnSOD Val16Ala polymorphism.

The association between manganese and T2D is likely complex because manganese is both an essential nutrient and a potential toxicant, depending on the amount of exposure. Similarly, our results suggested that both low and high levels of manganese were associated with increased risk of T2D, which was consistent in all stratified subgroups. Prior studies have reported conflicting results for the relationships between manganese and T2D. A recent study in Korea reported that the prevalence of self-reported diabetes increased significantly in participants in the lowest quartile for blood manganese (Koh et al. 2014). However, in that study, high blood manganese levels were found to be consistently associated with high blood pressure, but not with diabetes. Two previous case–control studies also indicated that diabetic patients had lower blood levels of manganese than controls in other populations (Forte et al. 2013; Kazi et al. 2008; Koh et al. 2014), and diabetic individuals showed no elevation of manganese levels compared with controls in other studies (Ekmekcioglu et al. 2001; Rambousková et al. 2013). In contrast, Anetor et al. found that serum manganese in patients with diabetes was double that in nondiabetic participants (Anetor et al. 2007). Additionally, significant interactions were found between plasma manganese concentration and age as well as physical activity in the present study; these findings have not been reported previously and remain to be validated in other studies.

The inconsistent findings between this study and previous studies might be related to the large sample size and the wide range of manganese levels in this study, which allowed us to examine the association of both low and high levels of manganese with T2D. In our study, the medians (IQRs) of plasma manganese concentration were 5.26 μg/L (3.67–8.33) for NGT and 4.37 μg/L (2.73–7.62) for T2D, higher than reference values (0.79 ± 0.63 μg/L) reported for adults by the Agency for Toxic Substances Registry (ATSDR 2012); these values were actually based on only one study with a small sample (*n* = 68) (Rükgauer et al. 1997). Several subsequent studies evaluated plasma manganese levels in healthy and diabetic individuals, but the results varied widely among studies. A study in Nigeria found that plasma manganese was significantly elevated in patients with diabetes (209 ± 0.39 μg/L) compared with healthy controls (99 ± 0.28 μg/L) (Anetor et al. 2007), but another study in Austria showed a nonsignificant elevation of plasma manganese in diabetic individuals compared with controls (1.81 ± 1.38 μg/L vs. 1.57 ± 0.98 μg/L) (Ekmekcioglu et al. 2001). At present, there is no internationally acceptable value or range for plasma manganese concentration in the general population. The discrepancies between populations remain to be elucidated because plasma manganese concentrations could be affected by exposure levels, effects of genetic predisposition and other predisposing factors on its metabolism, between-laboratory differences in methods (ICP-MS vs. electrothermal atomic absorption spectrometry) and measurement errors, and variations in population characteristics among studies. Physical activity was reported to be a widely accepted lifestyle factor in the prevention of T2D (Aune et al. 2015; Jeon et al. 2007), and it was found to be an important factor in manganese metabolism through generating large numbers of reactive oxygen species (ROS) (Watson 2014), which might explain the interaction between plasma manganese concentration and physical activity in the present study. Meanwhile, the effects on manganese metabolism of age and chronic manganese toxicity might explain the interaction of age with the Mn-T2D association (Aschner and Aschner 2005; Burton and Guilarte 2009). Owing to limited sample size and a limited study design, no previous study has been undertaken to investigate the interactions. Further studies with large samples, in particular prospective studies, are warranted to confirm the association between manganese levels and T2D.

The U-shaped association between plasma manganese and T2D is biologically plausible. Firstly, levels of manganese could affect the metalation and activity of MnSOD (Lee et al. 2013; Paynter 1980). Suboptimal MnSOD related to insufficient levels of manganese could result in increased mitochondrial ROS formation, which may directly cause macromolecular damage or might indirectly result in oxidative stress by activating stress-sensitive pathways such as the NFκB, p38 MAPK, JNK/SPAK, and hexosamine pathways (Evans et al. 2003). Activation of these pathways has been shown to lead to significant deterioration of glucose-stimulated insulin secretion (GSIS), mitochondrial dysfunction, and β-cell dysfunction (Anderson et al. 2009; El Khattabi and Sharma 2013; Hirosumi et al. 2002; Kamata et al. 2005). Accordingly, manganese supplementation may enhance MnSOD activity and protect against diabetes by enhancing insulin secretion (Burlet and Jain 2013; Lee et al. 2013). However, excessive manganese could lead to increased levels of MnSOD, selectively decreasing superoxide ion (O_2_^·^^–^) levels at the expense of increased hydrogen peroxide (H_2_O_2_) production. Transient exposure of β cells to 200 μM H_2_O_2_ may decrease the secretory response to glucose accompanied by β-cell dysfunction (Li et al. 2009), and long-term exposure to high levels of H_2_O_2_ may also lead to insulin resistance (Anderson et al. 2009; Bashan et al. 2009). Peroxisome-generated H_2_O_2_ has been shown to be involved in sensing signals that disrupt β-cell function (Elsner et al. 2011), and mitochondrial H_2_O_2_ emission was proposed to be a regulator linking excess fat intake to insulin resistance in the skeletal muscle of both rodents and humans (Anderson et al. 2009).

Secondly, independent of MnSOD, manganese supplementation was found to down-regulate ROS in both *in vitro* and *in vivo* studies (Burlet and Jain 2013), suggesting that there might be another mechanism involving manganese related to diabetes. However, manganese itself is an oxidant at high levels. Neurotoxicity from manganese overexposure appears to involve oxidative damage to dopaminergic neurons in particular, as well as mitochondrial dysfunction (Burton and Guilarte 2009; Desole et al. 1994; Dobson et al. 2003). It was shown that excessive manganese could compete with magnesium binding sites in proteins (Mukhopadhyay and Linstedt 2011), which also enhances oxidative stress. Thirdly, manganese is an essential mineral nutrient for glucose metabolism and other functions that directly activates certain enzymes or acts indirectly through other manganese-dependent metalloenzymes (Aschner and Aschner 2005). However, excess manganese can induce adverse effects, particularly in the central nervous system (Burton and Guilarte 2009; Sidoryk-Wegrzynowicz and Aschner 2013), which is also implicated in glucose homeostasis and diabetes (Schwartz et al. 2013). Finally, it has been postulated that manganese accelerates cellular glucose uptake by potentiating insulin action and that manganese may act on the pancreas by stimulating the release of stored insulin into the bloodstream or by inhibiting the release of glucagon (Rubenstein et al. 1962). However, more studies are warranted to clarify the mechanisms underlying the association between manganese and diabetes.

Because MnSOD is a major mitochondrial antioxidant and plays a critical role in protecting mitochondria and islets from ROS, many studies have elucidated the role of MnSOD and its encoding gene in relation to T2D and diabetes complications (Bresciani et al. 2013). The most commonly studied MnSOD polymorphism is Val16Ala because substitution of the T allele for the C allele leads to translation of the amino acid valine (GTT) instead of alanine (GCT). This change leads to a 30–40% reduction of MnSOD activity and to less efficient transport of MnSOD into the mitochondrial matrix (Sutton et al. 2003). However, the sample sizes of previous epidemiologic studies were rather small, and the results have been inconsistent (Bresciani et al. 2013; Flekac et al. 2008; Lee and Choi 2006; Liu et al. 2009; Nakanishi et al. 2008). A meta-analysis indicated a significant protective effect of the C allele on the risk of T2D (Tian et al. 2011), but this association disappeared after excluding one study that deviated from Hardy–Weinberg equilibrium (Flekac et al. 2008). In the present study, the MnSOD Val16Ala polymorphism was not associated with T2D. To our knowledge, this is the largest population-based study to comprehensively investigate the association between the Val16Ala polymorphism and T2D. Moreover, we found that the MnSOD genotypes did not modify the association between plasma levels of manganese and T2D risk. ROS are considered to be an important trigger of T2D, but T2D is also hypothesized to be accelerated or even caused by shortages in cellular ROS (Watson 2014). Although it is acknowledged that elevated MnSOD could protect against diabetes by down-regulating ROS in β cells and enhancing insulin secretion, some debate remains regarding whether increased MnSOD expression has beneficial or deleterious effects on muscle insulin sensitivity (Anderson et al. 2009; Hoehn et al. 2009). The frequency of the Ala (C) allele was rather low (~14%) in the present study, so there remains a need to validate the association between the MnSOD Val16Ala polymorphism and T2D, as well as the interaction with manganese, in large prospective studies.

The strengths of our study include the large number of participants and objectively measured plasma manganese levels. Our study participants with T2D were confined to newly diagnosed and drug-naïve patients because anti-diabetics or drugs may alter the status of manganese metabolism. Moreover, we defined diabetes based mainly on fasting and postprandial glucose levels from an OGTT.

Our study also has several limitations. First, the case–control nature of our study does not allow us to infer any causality between plasma manganese and T2D because plasma manganese levels may be affected by the development of insulin resistance and T2D. Second, our measurement of manganese was confined to the plasma compartment. We used plasma manganese as a biomarker to measure manganese status to avoid potential bias through dietary assessment, such as systematic measurement error in self-reported dietary exposure and the influence of other nutrients on the bioavailability of manganese (Davis et al. 1992; Finley 1999). Third, although we controlled for multiple T2D risk factors including several oxidative stress–related minerals (copper, selenium, iron) in plasma, we could not rule out the possibility that other correlated nutrients also contributed to the observed association. We also lacked information on the education level of the participants, plasma zinc levels, and inflammatory markers that might also have confounded our results. In addition, all participants in this study were of Chinese Han ethnicity, which minimizes the confounding effects by ethnic background but may limit the generalizability of the results to other ethnic groups. Furthermore, we did not measure the concentrations of manganese according to different valence states, which may have different effects on ROS formation, lipid peroxidation, and ensuing cell death (HaMai et al. 2001).

## Conclusions

Our study revealed a U-shaped association between plasma manganese concentrations and T2D in a Chinese population, and the association was not modified by the MnSOD Val16Ala polymorphism. Additional studies are warranted to confirm our findings in prospective cohorts and to elucidate the potential mechanisms underlying the relationship between manganese and T2D.

## Supplemental Material

(227 KB) PDF
