# Supplementary material for: U-Shaped Association between Plasma Manganese Levels and Type 2 Diabetes
Source: Environ Health Perspect. 2016 Jun 3;124(12):1876–81. doi: 10.1289/EHP176 (PMC5132633; doi:10.1289/EHP176)
Supplement: (227 KB) PDF [file EHP176.s001.acco.pdf]

Environ Health Perspect
DOI: 10.1289/EHP176

Note to readers with disabilities: *EHP* strives to ensure that all journal content is accessible to all readers. However, some figures and Supplemental Material published in *EHP* articles may not conform to 508 standards due to the complexity of the information being presented. If you need assistance accessing journal content, please contact ehp508@niehs.nih.gov. Our staff will work with you to assess and meet your accessibility needs within 3 working days.

Supplemental Material

U-Shaped Association between Plasma Manganese Levels and Type 2 Diabetes

Zhilei Shan, Sijing Chen, Taoping Sun, Cheng Luo, Yanjun Guo, Xuefeng Yu, Wei Yang, Frank B. Hu, and Liegang Liu

Table of Contents

Table S1 Association of the MnSOD Val16Ala polymorphism and T2D.

Table S2 ORs for the association between the MnSOD Val16Ala polymorphism and T2D in subgroups.

Table S3 ORs for the association between T2D and plasma manganese concentrations, according to the MnSOD Val16Ala polymorphism genotypes.

Table S1 Association of the MnSOD Val16Ala polymorphism and T2D.

	**Controls, n (%)**	**Cases, n (%)**	**Crude OR (95% CI)**	**P value**	**Adjusted OR[a] (95% CI)**	**P value**
Genotypes						
TT	1206(74.72)	1184(73.36)	1		1	
CT	378(23.42)	392(24.29)	1.06(0.90-1.24)	0.51	1.03(0.85-1.25）	0.77
CC	30(1.86)	38(2.35)	1.29(0.79-2.10)	0.3	1.07(0.60-1.92）	0.81
CT+CC	408(25.28)	430(26.64)	1.07(0.92-1.26)	0.38	1.03(0.85-1.25）	0.74
TT+CT	1584(98.14)	1576(97.65)	1		1	
CC	30(1.86)	38(2.35)	1.27(0.78-2.07）	0.33	1.07(0.60-1.90）	0.83

[a] adjusted for age, sex, BMI, current smoking status, current alcohol drinking status, physical activity, family history of diabetes and hypertension.

Environ Health Perspect
DOI: 10.1289/EHP176

Table S2 ORs for the association between the MnSOD Val16Ala polymorphism and T2D in subgroups.

Groups	MnSOD Val16Ala genotype			P value for interaction
	TT	CT	CC	
**Levels of Mn**				
Low	1	1.23(0.89-1.69）	0.79(0.34-1.84）	
Moderate	1	0.99(0.69-1.43）	0.90(0.32-2.52）	0.54
High	1	0.88(0.60-1.31）	2.81(0.55-14.38）	
**Sex**				
Male	1	1.06(0.82-1.38）	0.95(0.42-2.16）	0.35
Female	1	0.95(0.70-1.30）	1.48(0.63-3.51）	
**Age**				
< 55	1	1.11(0.79-1.56）	0.89(0.29-2.67）	0.55
≥ 55	1	1.00(0.78-1.28）	1.12(0.57-2.21）	
**BMI**				
< 24	1	0.91(0.69-1.21）	0.79(0.33-1.88）	0.42
≥ 24	1	1.13(0.85-1.50）	1.37(0.60-3.11）	
**Physical activity**				
No or rare	1	1.08(0.85-1.37）	1.00(0.50-1.99）	0.06
Yes	1	0.90(0.63-1.29）	1.30(0.44-3.85）	

Adjusted for age, sex, BMI, current smoking status, current alcohol drinking status, physical activity, family history of diabetes and hypertension.

Table S3 ORs for the association between T2D and plasma manganese concentrations, according to the MnSOD Val16Ala polymorphism genotypes.

Variables	Tertiles of plasma manganese concentration			P value for interaction
	1 (Lowest)	2	3(Highest)	
**TT**				
N(NGT/T2D)	410/575	386/272	410/337	
OR	1.82(1.43-2.32）	1	1.68(1.28-2.21）	
**CT**				
N(NGT/T2D)	115/188	142/95	121/109	**0.54**
OR	2.19(1.42-3.38）	1	1.57(0.97-2.53）	
**CC**				
N(NGT/T2D)	13/17	13/10	4/11	
OR	2.59(0.48-14.06）	1	4.52(0.47-43.3）	
**CT&CC**				
N(NGT/T2D)	128/205	115/105	125/120	**0.84**
OR	2.41(1.61-3.61)	1	1.59(1.02-2.49)	

Adjusted for age, sex, BMI, current smoking status, current alcohol drinking status, physical activity, family history of diabetes, hypertension, plasma iron, plasma copper, and plasma selenium.
